# The Enigmatic Role of GBA2 in Controlling Locomotor Function

**DOI:** 10.3389/fnmol.2017.00386

**Published:** 2017-11-28

**Authors:** Marina A. Woeste, Dagmar Wachten

**Affiliations:** ^1^Institute of Innate Immunity, University Hospital, University of Bonn, Bonn, Germany; ^2^Molecular Physiology, Center of Advanced European Studies and Research, Minerva Max Planck Research Group, Bonn, Germany

**Keywords:** beta-glucosidase, GBA2, glucosylceramide, glycosphingolipids, locomotor function

## Abstract

The non-lysosomal glucosylceramidase GBA2 catalyzes the hydrolysis of glucosylceramide to glucose and ceramide. Loss of GBA2 function results in accumulation of glucosylceramide. Mutations in the human *GBA2* gene have been associated with hereditary spastic paraplegia (HSP) and autosomal-recessive cerebellar ataxia (ARCA). Patients suffering from these disorders exhibit impaired locomotion and neurological abnormalities. *GBA2* mutations found in these patients have been proposed to impair GBA2 function. However, the molecular mechanism underlying the occurrence of mutations in the *GBA2* gene and the development of locomotor dysfunction is not well-understood. In this review, we aim to summarize recent findings regarding mutations in the *GBA2* gene and their impact on GBA2 function in health and disease.

## Introduction

Lipids are major constituents of cellular membranes in archaea, bacteria, and eukaryotes. Cellular membranes not only fulfill a barrier function to the environment, but also provide a platform for cellular processes. Various lipid species with different physical properties form functionally and structurally distinct lipid layers, which change dynamically and, in turn, determine the functional output of a cell. Defects in the lipid homeostasis alter cellular functions, resulting in severe diseases, e.g., neurological dysfunction. Among them are the autosomal-recessive cerebellar ataxia (ARCA) and hereditary spastic paraplegia (HSP), which share common symptoms with cerebellar signs typical for ataxia and spasticity and show a disease onset in early childhood. Furthermore, the underlying genes, cellular pathways, and disease mechanisms seem to be common (Synofzik and Schüle, 2017). For both, ARCA and HSP, patients with mutations in the human *GBA2* gene have been identified (Hammer et al., 2013; Martin et al., 2013; Citterio et al., 2014; Votsi et al., 2014; Sultana et al., 2015; Table 1). GBA2 is a beta-glucosidase, which degrades the glycosphingolipid glucosylceramide (GlcCer) to glucose and ceramide. Loss of GBA2 function results in accumulation of GlcCer and dysregulation of the lipid homeostasis (Raju et al., 2015; Schonauer et al., 2017). However, the molecular mechanism underlying the occurrence of mutations in the *GBA2* gene and the development of locomotor dysfunction is not well-understood. Here, we summarize the recent findings of GBA2-dependent control of GlcCer homeostasis and its role in controlling motor function in health and disease.

**Table 1:** Mutations in the human *GBA2* gene associated with locomotor dysfunction.

	**Mutation**	**Alleles**	**Human GBA2**	**Mouse GBA2**	**Associated disease**	**References**
Variants with an amino-acid substitution	2618G>A	homozygous	R873H	R864H	Autosomal-recessive cerebellar ataxia	Hammer et al., 2013
	2201G>A	Homozygous	R734H	R725H	Autosomal-recessive cerebellar ataxia	Votsi et al., 2014
	2048G>C	Homozygous	G683R	G674R	Hereditary spastic paraplegia	Citterio et al., 2014
	1888C>T	Homozygous	R630W	R621W	Hereditary spastic paraplegia	Martin et al., 2013
	1780G>C	Homozygous	D594H	D585H	Autosomal-recessive cerebellar ataxia	Votsi et al., 2014
	1255T>G	Heterozygous, co-segregated with 2608C>T	F419V	F410V	Hereditary spastic paraplegia	Sultana et al., 2015
Truncated variants	2608C>T	Heterozygous, co-segregated with 1255T>G	R870^*^	Q861^*^	Hereditary spastic paraplegia	Sultana et al., 2015
	1528_1529del	homozygous	M510V^*^16	M501A^*^16	Marinesco-Sjögren-Like Syndrome	Haugarvoll et al., 2017
	1471_1474dupGGCA	Heterozygous, co-segregated with 518G>A	T492R^*^9	T483R^*^9	Hereditary spastic paraplegia	Martin et al., 2013
	1017C>T	Homozygous	R340^*^	R331^*^	Autosomal-recessive cerebellar ataxia	Hammer et al., 2013
	700C>T	Homozygous	R234^*^	R225^*^	Hereditary spastic paraplegia	Martin et al., 2013
	518G>A	Heterozygous, co-segregated with 1471_1474dupGGCA	W173^*^	W164^*^	Hereditary spastic paraplegia	Martin et al., 2013
	363C>A	Homozygous	Y121^*^	Y112^*^	Autosomal-recessive cerebellar ataxia	Hammer et al., 2013

* *Indicates a stop codon*.

### Glucosylceramide metabolism and GBA2 function

**Figure 1:**
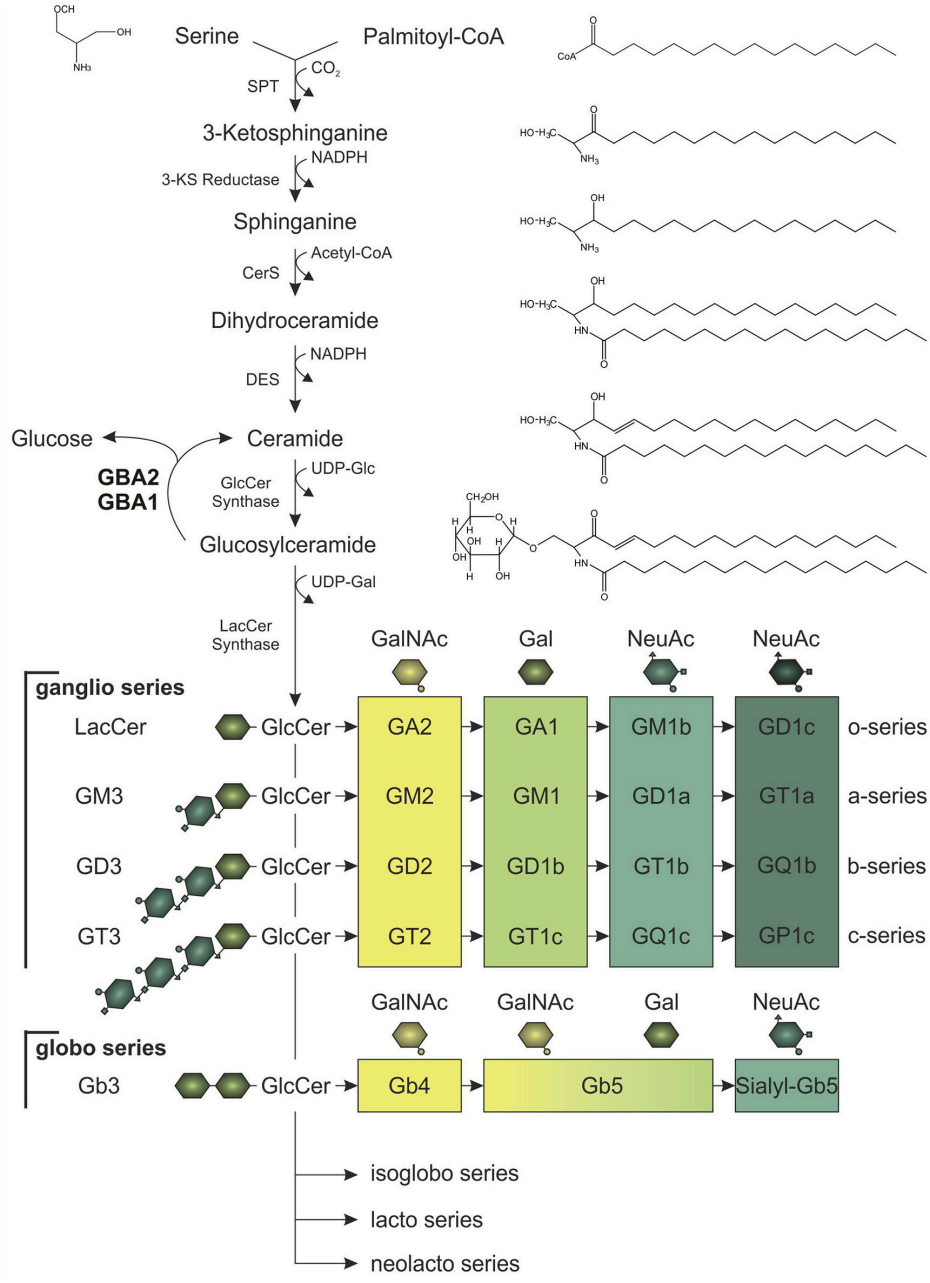
Synthesis of glucosylceramide (GlcCer) and its complex glycoshingolipid metabolites. In the *de novo* pathway, the serine palmitoyltransferase (SPT) catalyzes the synthesis step to form ceramide, which is the lipid backbone of GlcCer. Serine and palmitoyl-CoA are condensated to 3-ketosphinganine (3-KS). Subsequent reduction of 3-KS to sphinganine and acetylation by ceramide synthases (CerS) forms dihydroceramide. Desaturation catalyzed by ceramide desaturase (DES) yields ceramide, which can be glycosylated to GlcCer. After addition of galactose (Gal), GlcCer gives rise to more complex glycosphingolipids of the ganglio, globo, isoglobo, lacto, and neolacto series. To generate glycosphingolipids of the ganglio series, zero, one, two, or three N-acetylneuraminic acid residues (NeuAc) are attached (o-, a-, b-, or c-series). Galactosylation of lactosylceramide (LacCer; Gal-GlcCer) is necessary to build lipids of the globo series. For both ganglio and globo series, consecutive addition of N-acetylgalactosamine (GalNAc), Gal, and NeuAc by specific glucosyltransferases results in glycosphingolipids of different structural complexity and function. Glycosphingolipid homeostasis is highly dependent on GlcCer synthesis and its hydrolysis to ceramide and glucose, which is catalyzed by the lysosomal and non-lysosomal glucosylceramidase, GBA1 and GBA2, respectively.

The three major membrane lipid species are phospholipids, cholesterol, and sphingolipids (van Meer et al., 2008). Of these three, sphingolipids are the most complex lipid species. Ceramide is the building block to generate glycosylated sphingolipids known as glycosphingolipids. Glycosphingolipids are tremendously heterogeneous and vary (1) in the length of the fatty acid linked to the sphingosine molecule, (2) in the hydroxylation and saturation state of the fatty acid, and (3) in the oligosaccharide attached to the lipid backbone (Stults et al., 1989). Altogether, glycosphingolipids comprise more than 300 different lipids in mammals. The simplest glycosphingolipids galactosylceramide (GalCer) and GlcCer are synthesized by specific galactosyl- and glucosyltransferases that use UDP-galactose or UDP-glucose, respectively, and link the sugar moiety to ceramide via an O-glycosidic bond (Figure 1; Basu et al., 1968; Morell et al., 1970; Ichikawa et al., 1996). GlcCer is the precursor for more complex glycosphingolipids: globosides contain more sugar side-chains attached to ceramide, whereas gangliosides contain additional sialic acid moieties linked to ceramide. These complex glycosphingolipids are important structural components of the membrane, since they cluster in specific microdomains known as lipid rafts, and build a platform to gather certain proteins, e.g., ion channels and receptors (Simons and Toomre, 2000; Dart, 2010). Thus, glycosphingolipids play a key role in controlling cellular signaling, and regulating the levels of the glycosphingolipid precursor, GlcCer, is crucial to maintain cellular signaling. Degradation of GlcCer is catalyzed by glucosylceramidases, namely the lysosomal GBA1, also called glucocerebrosidase, the non-lysosomal membrane-associated GBA2, and the cytosolic Klotho-related glucosylceramidase GBA3, with the latter playing only a minor role in glycosphingolipid homeostasis (Ho and O'Brien, 1971; Matern et al., 1997; Hayashi et al., 2007). These glucosylceramidases hydrolyze GlcCer to release the monosaccharide glucose from the ceramide backbone (Figure 1). GBA1 activity was not only the first to be identified, but is also the one that has been most comprehensively studied. It conveys the main hydrolysis of GlcCer in the lysosome and mutations in the *GBA1* gene result in a severe lysosomal storage disorder called *Gaucher* disease (Brady et al., 1965; Grace et al., 1994). Only in the last 10 years, the physiological function of GBA2 and its contribution to GlcCer degradation has been identified. The human *GBA2* gene is encoded on chromosome 9 and spans 17 exons. The GBA2 protein consists of 927 amino acids and is highly conserved among different species (87% sequence identity between human GBA2 and mouse GBA2). GBA2 is ubiquitously expressed, with highest expression levels found in liver, brain, and testis (van Weely et al., 1993; Matern et al., 1997). The enzyme was initially identified as a bile-acid β-glucosidase (Matern et al., 1992), but later shown to cleave GlcCer (Matern et al., 1992; Boot et al., 2007). Although GBA1 and GBA2 share the same substrate GlcCer, they do not show any sequence homology, and are localized in different cellular compartments: GBA1 is localized in the lysosome, whereas GBA2 activity has been first associated with the plasma membrane (Aureli et al., 2009, 2011); however, reports analyzing its cellular localization demonstrate that GBA2 is a membrane-associated protein at the cytoplasmic site of the ER and Golgi membrane (Körschen et al., 2013). The two activities can be distinguished by their different pH optima (GBA1: pH 4–4.5, GBA2: pH 5.5–6; van Weely et al., 1993; Aureli et al., 2009; Körschen et al., 2013). Furthermore, GBA2 can be pharmacologically blocked by iminosugars [*N*-deoxynojirimycin (AMP-DNM), *N*-butyldeoxynojirimycin (NB-DNJ) and *N*-butyldeoxygalactonojirimycin (NB-DGJ)] that mimic the cyclic saccharide, but lack an O-glycosidic bond for enzyme cleavage (Ridley et al., 2013). GBA2 not only hydrolyzes GlcCer, it also exhibits a transglucosylation activity, transferring glucose to cholesterol. Thus, the glucose moiety released by the cleavage of GlcCer can be used by GBA2 to form glucocholesterol (GlcChol). Vice versa, GlcChol can be deglycosylated by GBA2 to synthesize GlcCer (Marques et al., 2016).

Loss of GBA2 results in the accumulation of GlcCer, in particular in those tissues where GBA2 expression is highest, i.e., testis, brain, and liver (Yildiz et al., 2006). The predominant phenotype associated with the genetic loss of GBA2 in a mouse model was observed in male reproduction: male GBA2 knockout mice were subfertile due to a sperm morphological defect occurring during spermatogenesis, a condition called globozoospermia (Yildiz et al., 2006). A similar phenotype was observed when GBA2 was pharmacologically inhibited (van der Spoel et al., 2002; Walden et al., 2007). On a molecular level, this phenotype was shown to be caused by the accumulation of GlcCer in sperm and Sertoli cells, which alters the lipid stacking of the plasma membrane and, in turn, dysregulates the cytoskeletal dynamics, impairing sperm development (Yildiz et al., 2006; Raju et al., 2015). Furthermore, GBA2 knockout mice also displayed a defect in liver regeneration when undergoing partial hepatectomy (Gonzalez-Carmona et al., 2012), which seems to be due to an IL-6-dependent change in the STAT3 signaling cascade (Gonzalez-Carmona et al., 2012). However, the distinct role of GBA2 in the brain, where its expression level is also high, has not been identified. Whereas mutations in the *GBA2* gene in human patients have been associated with impaired locomotion and neurological abnormalities, the analysis of GBA2-knockout mouse models did not reveal neurological symptoms or a defect in locomotor function yet.

### The role of glycosphingolipids in the central nervous system

Glycosphingolipids - especially gangliosides - are highly abundant in neurons, where they undergo constant changes in their localization pattern and overall content in the plasma membrane (Aquino et al., 1987). Ganglioside content in the brain increases during development, more precisely the amount of more complex species of the a- and b-series (Figure 1; Svennerholm et al., 1989). Fluctuation in the expression of the distinct glucosyltransferases and glucohydrolases allows the cells to adapt to differentiation processes (Aureli et al., 2011). In fact, GBA2 expression increases during neuronal differentiation (Aureli et al., 2012). Morphological changes during neuronal differentiation and axon growth are also accompanied by changes in the lipid content of the cell (Aureli et al., 2014). Due to their structural asymmetry with the hydrophobic ceramide inserted into the external leaflet of the membrane and the oligosaccharide facing the extracellular space, gangliosides play a crucial role in restructuring of the membrane: The complex hydrophilic oligosaccharide moieties induce a spatial segregation of the lipids across the membrane, whereby a positive membrane curvature is generated (Brocca and Sonnino, 1997). Moreover, lipid separation promotes the formation of microdomains, so-called lipid rafts, consisting of gangliosides and cholesterol (Simons and Toomre, 2000). Loss of glycosphingolipid synthesis in neurons leads to severe cellular defects: inhibition of ceramide synthesis in hippocampal neurons and Purkinje cells impairs axon and dendritic growth, respectively (Harel and Futerman, 1993; Furuya et al., 1995). Furthermore, GlcCer synthesis was demonstrated to be a prerequisite for growth factor bFGF- and laminin-induced axon growth (Boldin and Futerman, 1997). *In vivo*, perturbation of the glycosphingolipid homeostasis leads to neurological dysfunctions. Knockout mouse-models of distinct synthases of the ganglio series resembled comparable phenotypes: Loss of gangliosides in GM2/GM3 synthase double-knockout mice led to severe ataxia and limb weakness due to axon degeneration (Yamashita et al., 2005). Mice devoid of the GM2/GD2 and GD3 synthase also revealed gait abnormalities in line with tremor, resulting from neurodegeneration affecting the cerebellum. Knockout of GM2/GD2 synthase alone also caused neuropathy in mice (Chiavegatto et al., 2000; Ohmi et al., 2009). Thus, maintenance of glycosphingolipid homeostasis is required for proper neuron function.

### Mutations in the *GBA1* or *2* gene associated with neurological disorders

Mutations in the *GBA1* gene have been associated with Parkinson's disease (PD), the second most common neurodegenerative disorder after Alzheimer's disease, characterized by slow movements accompanied by decrement and degradation of repetitive movements (Mitsui et al., 2009; Sidransky et al., 2009; Shachar et al., 2011; Blanz and Saftig, 2016; Migdalska-Richards and Schapira, 2016). Homozygous mutations in *GBA1* cause *Gaucher* disease, but an increased risk in developing PD has not only been observed for *Gaucher* disease patients, but already in heterozygous carriers (Migdalska-Richards and Schapira, 2016). Carriers of a *GBA1* mutation contain an up to 30-fold higher risk of developing PD and at least 7–10% of PD patients carry a *GBA1* mutation (Migdalska-Richards and Schapira, 2016). However, the underlying molecular mechanism is still ill-defined. Different hypotheses have been proposed how *GBA1* mutations promote the development of PD. PD belongs to a group of diseases commonly referred to as synucleinopathies, which are characterized by the presence of Lewy bodies and α-synuclein in neurites. A shift of α-synuclein from the monomeric to the oligomeric toxic and aggregated form has been shown to underlie PD progression (Blanz and Saftig, 2016; Migdalska-Richards and Schapira, 2016). One model describes a direct interaction between the mutated GBA1 protein and α-synuclein, leading to α-synuclein accumulation and aggregation (Sidransky and Lopez, 2012). Another model proposes that decreased GBA1 activity, leading to lysosomal dysfunction, and accumulation of GlcCer and its related lipid metabolites affect α-synuclein trafficking, processing, and clearance, promoting α-synuclein aggregation and oligomer formation (Sidransky and Lopez, 2012; Blanz and Saftig, 2016; Migdalska-Richards and Schapira, 2016). Based on these models, strategies aiming to increase either the expression, stability, or delivery of GBA1 to the lysosomes are likely to decrease the α-synuclein burden, and may be used as a treatment for PD.

**Figure 2:**
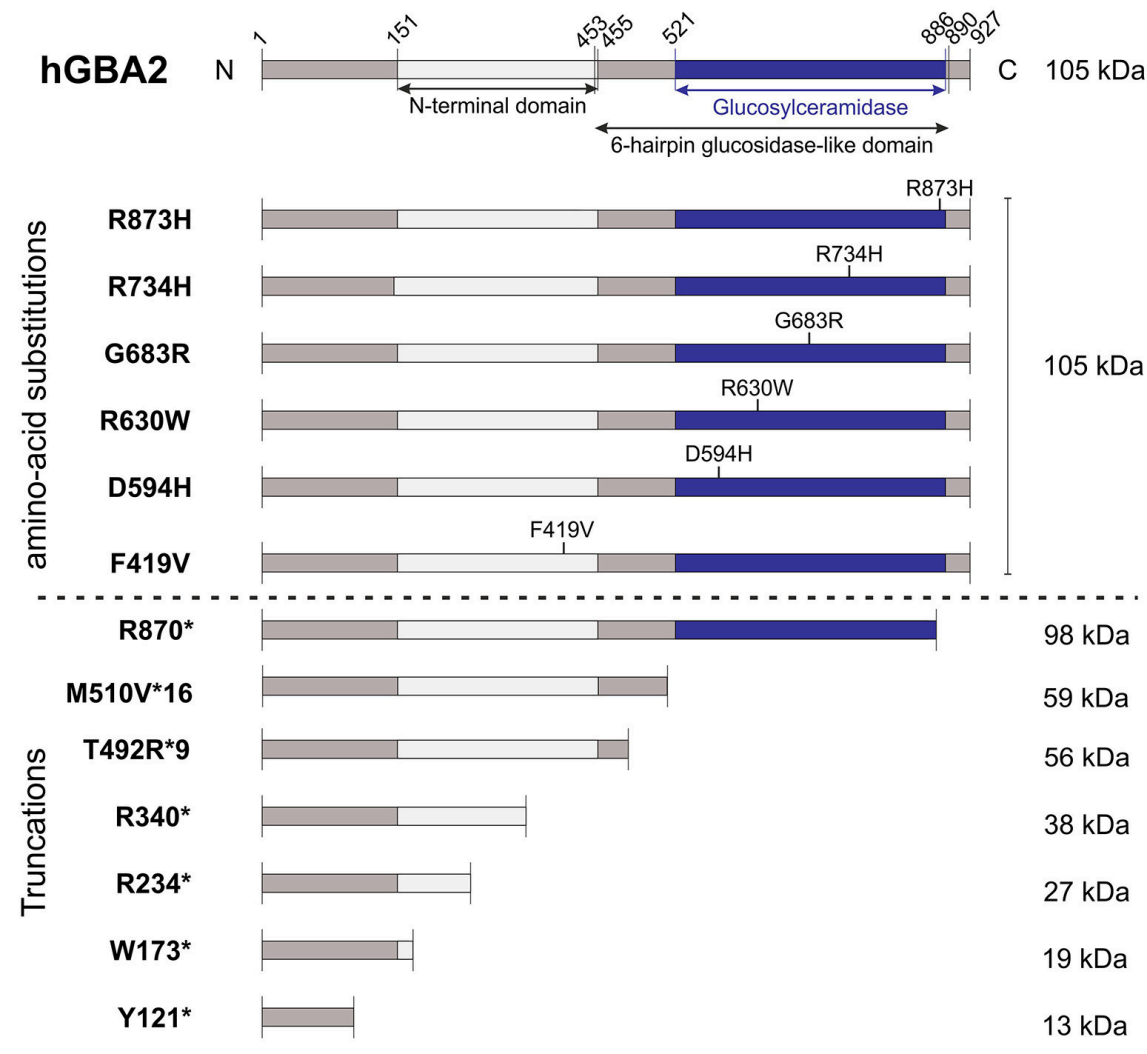
Domain structure hGBA2 wild-type vs. mutants. The domain structure of hGBA2, annotated according to InterPro (Finn et al., 2017), is indicated. Mutations found in human patients with ARCA, HSP, or Marinesco-Sjögren-Like Syndrome are shown. The molecular weight of the corresponding hGBA2 proteins is indicated on the right. ^*^Indicates a stop codon.

The correlation between mutations in the *GBA2* gene and movement disorders is less well-characterized. ARCA patients suffer from problems in balance and limb coordination, dysarthria, increased tone in the limbs and often, in later stages, pronounced limb spasticity (Fogel and Perlman, 2007). In 2013, homozygosity mapping and whole-exome sequencing results of three Tunisian families, including seven patients suffering from ARCA of unknown genetic origin, were published. Patients were initially diagnosed according to their predominant ataxic symptoms at disease onset. However, during disease progression, they also developed spasticity of the lower and upper limbs (Hammer et al., 2013). All of them harbored mutations in the *GBA2* gene: Two families contained a mutation in exon 5 (c.1017C>T), resulting in the truncated protein variant R340^*^, lacking the C-terminal beta-glucosidase domain (Figure 2). The third family carried a mutation in exon 17 (c.2618G>A), resulting in the amino-acid exchange R873H (Figure 2). For both mutations, only homozygous carriers were affected. Additional screening of 21 Tunisian individuals, suffering from cerebellar ataxia of unknown genetic cause, revealed a third mutation in exon 2 of the *GBA2* gene in three siblings (c.363C>A), resulting in a truncated GBA2 protein Y121^*^, lacking both the C-terminal beta-glucosidase and the N-terminal domain (Figure 2). All identified *GBA2* mutations were absent in 50 healthy Tunisian families and in 330 controls of the Human Genome Diversity Project (HGDP; Hammer et al., 2013). In 2014, two more *GBA2* mutations in three ARCA patients in a Cypriot family were identified using homozygosity mapping and exome sequencing of chromosome 9: mutations in exon 11 (c.1780G>C) and in exon 15 (c.2201G>A) resulted in the amino-acid substitution D594H and R734H, respectively (Figure 2; Votsi et al., 2014). Patients were diagnosed with spasticity of the lower limbs and cerebellar symptoms. Heterozygous carriers in the family were not affected, indicating that the mutations are only pathogenic in the homozygous state. None of these mutations were found in 52 control Cypriot individuals (Votsi et al., 2014).

HSP patients suffer from dysfunction or degeneration of motor neurons, resulting in locomotor dysfunction such as spasticity, brisk reflexes, and pyramidal weakness of the lower limbs (Harding, 1983; Schüle and Schöls, 2011). Multiple loci have been mapped to this largely heterogeneous group of disorders with spastic paraplegia 46 on chromosome 9, containing the *hGBA2* gene, being one of them. A number of mutations in the *GBA2* gene have been associated with HSP (Martin et al., 2013; Citterio et al., 2014; Sultana et al., 2015). Screening of 46 families of both Italian and African origin, suffering from complicated forms of HSP, revealed one patient with severe spasticity of the lower limbs and mild cerebellar signs carrying the mutation c.2048G>C in exon 13 on both alleles of the *GBA2* gene, resulting in the amino-acid exchange G683R (Figure 2). This gene variant was tested against 600 controls of Italian origin and the genome databases dbSNP and 1,000 Genomes, but only occurred in the affected subject (Citterio et al., 2014). Furthermore, one missense and three truncated GBA2 variants were identified in the genome of patients suffering from HSP accompanied by cerebellar ataxia (Martin et al., 2013). The amino-acid substitution R630W (1888C>T; Figure 2) in exon 12 was identified in a homozygous state in six individuals of two families, but in none of the 519 healthy controls (Martin et al., 2013). In a third family, the mutation c.700C>T in exon 4 was found in one HSP patient in a homozygous state, resulting in the truncated protein hGBA2 R234^*^, lacking the C-terminal beta-glucosidase domain and part of the N-terminal domain (Figure 2). The heterozygous mutation c.518G>A in exon 3 and c.1471_1474dupGGCA in exon 9, resulting in the truncated variants W173^*^ and T492R^*^9, respectively, co-segregated with the disease in another family (Martin et al., 2013). The truncated proteins lack either both the C-terminal beta-glucosidase and the N-terminal domain or only the C-terminal domain (Figure 2).

Recently, mutations in the *GBA2* gene have also been associated with the Marinesco-Sjögren-Like Syndrome, with patients showing both typical ARCA and HSP disease characteristics (Haugarvoll et al., 2017). Whole-exome sequencing revealed the homozygous mutation c.1528_1529del, resulting in the amino-acid substitution M510V^*^16 (Figure 2) in two Norwegian families and one unrelated affected subject, but not in any of the tested 500 control exomes nor 192 control blood samples (Haugarvoll et al., 2017). A summary of all mutations is presented in Table 1.

So far, only one mutation has been functionally characterized *in vivo* in an animal model. In a zebrafish model, GBA2 expression was severely reduced by injecting antisense *zGba2* mRNA into zebrafish larvae. This resulted in a malformed tail and a defect in motor coordination in 12.5% of injected animals (Martin et al., 2013). A more detailed behavioral analysis (touch-response test) showed that also injected zebrafish lacking the curly tail phenotype exhibited motor defects. Immunohistochemical analyses of motor neurons in the spine revealed a defect in axonal growth. Co-injection of human wild-type GBA2 mRNA rescued this axonal phenotype, whereas the mutant R630W failed to compensate for the loss of *zGBA2* (Martin et al., 2013). However, a detailed analysis of the different mutant GBA2 proteins and their physiological relevance in a mammalian animal model is still missing. First attempts have been made to characterize the different mutations in a heterologous expression system. The mutant GBA2 variants Y121^*^, W173^*^, R234^*^, R340^*^, F419V, R870^*^, D594H, R630W, G683R, and R873H all displayed reduced expression levels compared to wild-type GBA2 (Sultana et al., 2015). In a luciferase-based activity assay of transfected COS-7 and HeLa cells, all GBA2 mutants were null mutations without any activity compared to the wild-type GBA2 protein. Thus, human patients carrying these mutations might lack GBA2 activity (Sultana et al., 2015).

So far, it is not known how these mutations affect GBA2 function on a molecular level. The recently published crystal structure of a bacterial β-glucosidase helped to understand the consequence of *GBA2* mutations on protein function. The bacterial β-glucosidase TxGH116, expressed in *Thermoanaerobacterium xylanolyticum*, belongs to the same enzyme family GH116 as GBA2 (Charoenwattanasatien et al., 2016). The sequence encoding the catalytic domain of TxGH116 bears 40% sequence identity to GBA2. Amino-acid residue D508 and R786 corresponding to D594 and R873 in hGBA2, respectively, face the catalytic cleft of the enzyme, and are probably involved in forming hydrogen bonds with the 6-OH and 3-OH group of the glucose, respectively (Charoenwattanasatien et al., 2016). Thus, mutating one of these amino acids could directly interfere with the catalytic activity of GBA2. The amino acid G683 of hGBA2 seems to be located in a loop, which is important for the acid/base and the sugar binding. Mutations in this region might perturb loop formation and, thereby GBA2 activity. Furthermore, R630 in hGBA2 seems to undergo electrostatic interactions with the anionic carboxylate of E555 and D631, which are in close proximity. The loss of net charge at this position caused by an amino-acid substitution to tryptophan and the subsequent loss of amino-acid interaction might destabilize GBA2 (Charoenwattanasatien et al., 2016). Solving the crystal structure of a mammalian GBA2 protein will reveal whether the homology modeling according to the bacterial protein structure reliably predicts the structure of hGBA2 and allows to verify disease-causing mutations. So far, data suggest that the mutations found in human *GBA2* gene are loss-of-function mutations. Loss of GBA2 activity results in accumulation of GlcCer, which is supposed to change the lipid organization in the plasma membrane to a more ordered state and promote actin polymerization (Raju et al., 2015). The effect of GlcCer accumulation on membrane stacking and actin dynamics has been mainly studied in fibroblasts or germ cells during sperm development. Whether similar mechanisms occur in neurons, underlying the development of neuropathy in the absence of GBA2, is not known. Studies investigating the cellular phenotypes after loss of GBA1 activity, GBA2 activity, or both revealed that (a) there is a complex interplay between the two beta-glucosidases, and (b) that accumulation of GlcCer cannot be the sole determinant for the development of cellular pathologies (Mistry et al., 2014; Schonauer et al., 2017). In fact, concomitant loss of GBA2 activity in a GBA1-mouse model for *Gaucher* disease rescued the clinical phenotypes although glycosphingolipid levels were increased (Mistry et al., 2014). Furthermore, loss of GBA1 activity down-regulated GBA2 activity in fibroblasts from patients with *Gaucher* disease. This was conveyed through sphingosine, the cytotoxic metabolite accumulating in *Gaucher* cells through the action of GBA2: sphingosine bound to GBA2 and inhibited its activity (Schonauer et al., 2017). The role of sphingosine in the development of neuropathy in GBA2 knockout mice has not been investigated yet.

## Concluding remarks

Altogether, GBA2 function and homeostasis seem to be important for brain function and motor coordination. However, whether the defects are only occurring in neurons or also in skeletal muscle is not known. Furthermore, the underlying molecular mechanism is enigmatic. The analysis is hampered by the fact that in GBA2-knockout mouse models, neither neurological symptoms nor locomotor dysfunction has been observed yet. Thus, more detailed studies in mammalian animal models and state-of-the art biophysical and biochemical approaches will be needed to unravel the physiological function of GBA2 and GlcCer in the brain and their role in motor coordination in health and disease.

## Author contributions

All authors listed have made a substantial, direct, and intellectual contribution to the work, and approved it for publication.

### Conflict of interest statement

The authors declare that the research was conducted in the absence of any commercial or financial relationships that could be construed as a potential conflict of interest.
